# Array of Synthetic Oligonucleotides to Generate Unique Multi-Target Artificial Positive Controls and Molecular Probe-Based Discrimination of *Liposcelis* Species

**DOI:** 10.1371/journal.pone.0129810

**Published:** 2015-06-18

**Authors:** Mohammad Arif, George Opit, Abigail Mendoza-Yerbafría, Shefali Dobhal, Zhihong Li, Zuzana Kučerová, Francisco M. Ochoa-Corona

**Affiliations:** 1 National Institute for Microbial Forensics & Food and Agricultural Biosecurity, 127 Noble Research Center, Oklahoma State University, Stillwater, Oklahoma, 74078, United States of America; 2 Department of Entomology and Plant Pathology, 127 Noble Research Center, Oklahoma State University, Stillwater, Oklahoma, 74078, United States of America; 3 Department of Entomology, College of Agriculture and Biotechnology, China Agricultural University, Yuanmingyuan West Road 2, Beijing, 100193, PR China; 4 Crop Research Institute, Drnovská 507, 161 06, Prague, 6, Czech Republic; Naval Research Laboratory, UNITED STATES

## Abstract

Several species of the genus *Liposcelis* are common insect pests that cause serious qualitative and quantitative losses to various stored grains and processed grain products. They also can contaminate foods, transmit pathogenic microorganisms and cause allergies in humans. The common occurrence of multi-species infestations and the fact that it is difficult to identify and discriminate *Liposcelis* spp. make accurate, rapid detection and discriminatory tools absolutely necessary for confirmation of their identity. In this study, PCR primers and probes specific to different *Liposcelis* spp. were designed based on nucleotide sequences of the cytochrome oxidase 1 (CO1) gene. Primer sets ObsCo13F/13R, PeaCo15F/14R, BosCO7F/7R, BruCo5F/5R, and DecCo11F/11R were used to specifically detect *Liposcelis obscura* Broadhead, *Liposcelis pearmani* Lienhard, *Liposcelis bostrychophila* Badonnel, *Liposcelis brunnea* Motschulsky and *Liposcelis decolor* (Pearman) in multiplex endpoint PCRs, which amplified products of 438-, 351-, 191-, 140-, and 87-bp, respectively. In multiplex TaqMan qPCR assays, orange, yellow, red, crimson and green channels corresponding to reporter dyes 6-ROXN, HEX, Cy5, Quasar705 and 6-FAM specifically detected *L*. *obscura*, *L*. *brunnea*, *L*. *bostrychophila*, *L*. *pearmani* and *L*. *decolor*, respectively. All developed primer and probe sets allowed specific amplification of corresponding targeted *Liposcelis* species. The development of multiplex endpoint PCR and multiplex TaqMan qPCR will greatly facilitate psocid identification and their management. The use of APCs will streamline and standardize PCR assays. APC will also provide the opportunity to have all positive controls in a single tube, which reduces maintenance cost and labor, but increases the accuracy and reliability of the assays. These novel methods from our study will have applications in pest management, biosecurity, quarantine, food safety, and routine diagnostics.

## Introduction

Psocids (booklice or barklice) have caused problems as pests of stored grains and grain products for the last 50 years [1–2]. As far back as over one decade ago, psocids became recognized as serious stored-product pests in some parts of Australia [3]; in the United States they are recognized as pests of substance that cause grain weight loss by consuming the germ and endosperm [4–6]. Psocids are pests because they contaminate food and vector human pathogens [7–9], trigger allergies in humans [10], are not easily controlled by insecticides that are effective against other stored-product pests [11–16], and because the commodities infested by psocids can be rejected for export [6, 17].

Psocids, which belong to the genus *Liposcelis* (Insecta; Psocodea; Liposcelididae), are most frequently encountered infesting stored products [18]. Psocid infestations usually comprise more than one *Liposcelis* species [14, 19]. Furthermore, stored-product psocids are able to coexist for a long time on the commodity they infest but this phenomenon is related to species composition [20]

Management of *Liposcelis* species is difficult because of their differential response to commonly used insecticides and the fumigant phosphine [21–23]; this makes the correct identification of psocids extremely important for successful control. Identification and discrimination of *Liposcelis* species is done largely by morphological characterization. This task is complicated by the fact that adults are small (ca. 1 mm), and different species are morphologically similar. Discriminating among immature (nymphal) stages of *Liposcelis* is even more difficult. As a result, identification of both adult and immature stages is usually conducted by specialists or taxonomists, experts who are quite rare [6, 24–29]. Rapid and accurate methods for identifying *Liposcelis* species are needed for more effective integrated management.

The current availability of nucleic acid-based detection and identification technologies permits rapid, sensitive, and accurate discrimination among morphologically identical species. Conventional or standard endpoint PCR, SYBR Green qPCR and isothermal based methods for identification of psocids have been reported [30–33]. TaqMan probe based qPCR assays have been developed for highly accurate bioforensic detection/identification of plant pathogens [34–35]. However, the cost per reaction of TaqMan qPCR is slightly higher ($1.1) than that of SYBR green based assays ($0.93) [36]. To the best of our knowledge, there are no published studies on the use of multiplex TaqMan qPCR for identification of *Liposcelis* species.

Molecular assays can now be further enhanced using synthetic biology approaches, such as the development of clonable artificial systems for reproducing natural biological processes [37]. Development of multi-target artificial positive PCR controls (APCs) speeds molecular processing and improves biosafety [37]. Their use allows detection of cross contamination during PCR assays without compromising sensitivity and specificity. The APC approach is a unique way to amplify PCR diagnostic products of different sizes for clear discrimination, and has the advantage to allow rearrangement of the targets, periodic updates (insertion and deletion of new targets), monitoring quality control and international or regional exchange among research and diagnostic centers without the need for sanitary permissions or labels which are time consuming and hard to obtain from respective national and international authorities.

In this work, we developed accurate and rapid (short cycle) multiplex endpoint and multiplex TaqMan qPCR assays for the simultaneous identification and discrimination of five morphologically identical *Liposcelis* species, namely, *L*. *brunnea*, *L*. *decolor*, *L*. *bostrychophila*, *L*. *pearmani* and *L*. *obscura*. We also designed and developed customized synthetic PCR APCs. These tools provide capability for applications in insect diagnostics, discrimination and management, population monitoring and agricultural biosecurity.

## Materials and Methods

### Ethics statement

No specific permission from any government agency or relevant regulatory body was required for the collection of these insects at all of the sites listed below. Collection sites included private facilities that had insect-infested stored grain on site and for which the owners gave us full permission to collect insects. Other collection sites were university research or agricultural research facilities for which we had full permission to collect insects. None of the field sites or the collections of insects from them involved endangered or protected species.

### Insects

Eleven psocid species, *L*. *brunnea*, *L*. *decolor*, *L*. *bostrychophila*, *L*. *pearmani*, *L*. *obscura*, *Liposcelis entomophila* (Enderlein), *Liposcelis fusciceps* Badonnel, *Liposcelis rufa* Broadhead, *Liposcelis corrodens* Heymons, *Liposcelis paeta* Pearman, and *Lepinotus reticulatus* Enderlein were collected in 2004–2009 from different storage types and locations in the United States (Table 1); and reared on a mixture of 93% cracked hard red winter wheat, 5% Rice Krispies (Kellogg Company) and 2% wheat germ (wt/wt) [38] at the Oklahoma State University Stored Product Entomology Laboratory, Stillwater, OK. *Lepinotus inquilinus* von Heyden and *Lepinotus patruelis* Pearman were obtained from Illinois State University, Normal, IL, USA. Voucher specimens of 100 females each of *L*. *rufa*, *L*. *pearmani*, *L*. *bostrychophila*, *L*. *decolor*, *L*. *entomophila*, *L*. *paeta*, *L*. *brunnea*, *L*. *fusciceps*, *L*. *corrodens*, *L*. *obscura* and *L*. *reticulatus* preserved in 95% ethyl alcohol were deposited at the K. C. Emerson Entomology Museum at Oklahoma State University under lot numbers 101, 103, 106, 107, 110, 111, 114, 116, 118, 119 and 120, respectively [30].

**Table 1:** Details of psocid species.

Species	Collection locality	Storage type (commodity)	Year collected
*Liposcelis brunnea*	CGAHR Manhattan, KS, USA	Grain elevator (wheat)	2006
*Liposcelis decolor*	CGAHR Manhattan, KS, USA	Steel bins (wheat)	2006
*Liposcelis bostrychophila*	CGAHR Manhattan, KS, USA	Grain elevator (wheat)	2006
*Liposcelis pearmani*	SPREC Stillwater, OK, USA	Animal feed mill	2008
*Liposcelis obscura*	Golden, OK, USA	Warehouse (peanuts)	2009
*Liposcelis entomophila*	CGAHR Manhattan, KS, USA	Steel bins (wheat)	2004
*Liposcelis fusciceps*	West Lafayette, IN, USA	Animal feed mill	2006
*Liposcelis rufa*	SPREC Stillwater, OK, USA	Steel bins (wheat)	2008
*Liposcelis corrodens*	CGAHR Manhattan, KS, USA	Grain elevator (wheat)	2006
*Liposcelis paeta*	CGAHR Manhattan, KS, USA	Grain elevator (wheat)	2006
*Lepinotus reticulatus*	CGAHR Manhattan, KS, USA	Steel bins (wheat)	2004

Information on where different psocid species used in the study were collected.

CGAHR is Center for Grain and Animal Health Research; SPREC is the Stored Product Research and Education Center.

### DNA isolation

The identity of each species of psocids was confirmed using decisive morphological traits (Dr. E. Mockford confirmed the identity of all psocid species used in our study). Genomic DNA from 10–20 individuals of each species was extracted using the Blood and Tissue Kit (Qiagen, Valencia, CA). A prepGEM kit (ZyGEM Corporation Ltd, Hamilton, New Zealand) was used for rapid isolation of DNA from a single insect of each species. The DNeasy Plant Mini Kit (Qiagen) and QIAprep Spin Miniprep Kit (Qiagen) were used to isolate DNA from dry cracked wheat pieces (‘Jagger’ variety) and cloned plasmid DNA from overnight grown bacterial cultures carrying the target sequences of each of the respective species. All procedures were performed following the manufacturer’s instructions. DNA concentrations were determined using a NanoDrop v.2000 spectrophotometer (Thermo Fisher Scientific Inc., Worcester, MA).

### *Liposcelis* CO1 region amplification

The amplification of unknown CO1 regions (not available in GenBank) of *L*. *obscura*, *L*. *pearmani*, *L*. *bostrychophila* and *L*. *decolor* was approached by performing PCR with combinations of previously reported universal primers for insects [39–42]. Reagents, PCR conditions and electrophoresis for amplification of CO1 gene region of the above listed species using universal primer sets were previously described by Arif et al. [30]. The purification of amplified PCR products was accomplished using either Illustra GFX PCR DNA or the Gel Band Purification Kit (GE Healthcare Biosciences, Piscataway, NJ) following the manufacturer’s instructions. All PCR products were directly sequenced using an Applied Biosystems DNA Analyzer (Model # 3730) at the Oklahoma State University Nucleic Acids and Proteins Core Facility. Sense primers were: UEA1d, UEA1, LCO, Ron, and UEA3 and anti-sense primers were: Nancy, HCO, UEA6, UEA8 and UEA10. Sense and anti-sense of above primer combinations were made and tested across the COI gene region of the above species (S1 Fig). The primer sets Ron/UEA8 for *L*. *bostrychophila*, *L*. *pearmani* and *L decolor*, LCO1490/HCO2198 for *L*. *bostrychophila*, LCO1490/UEA6 for *L*. *obscura*, and LCO1490/Nancy for *L*. *pearmani*, were used for sense and anti-sense strand sequencing. The gene sequences generated using these universal primers combinations were submitted to GenBank under the accession numbers of KP012572, KP012571, KP012569 and KP012570.

### Species specific primer and probe design

The CO1 gene sequences of individual *Liposcelis* species obtained using universal sense and anti-sense primers were aligned using CLUSTALX2 [43] to generate the consensus sequence. Sense and anti-sense primers for multiplex endpoint and TaqMan qPCR with the respective probes for each species were designed within the newly obtained CO1 gene regions (Tables 2 and 3) using Primer3 [44], following parameters previously reported by Arif and Ochoa-Corona [45]. The primers and probe for *L*. *brunnea* were designed using accession GenBank: GU569291 (Tables 2 and 3). Prediction of internal structure and self-dimer formation of each primer and probe were examined *in silico* using Primer3 “ANY” score and the mFold output [46]. Primer sets ObsCo13F/ObsCo13R (product size 438 bp), PeaCo15F/PeaCo14R (product size 351 bp), BosCO7F/BosCO7R (product size 191 bp), BruCo5F/BruCo5R (product size 140 bp), and DecCo11F/DecCo11R (product size 87 bp) for *L*. *obscura*, *L*. *pearmani*, *L*. *bostrychophila*, *L*. *brunnea* and *L*. *decolor*, respectively, were designed to perform single and multiplex endpoint PCR amplifications (Table 2). A second group of primer sets BruCo5F/BruCo5R (140 bp), BosCo8F/BosCo8R (131 bp), PeaCo14F/PeaCo14R (115 bp), ObsCo12F/ObsCo12R (100 bp), and DecCo11F/DecCo11R (87 bp) for *L*. *brunnea*, *L*. *bostrychophila*, *L*. *pearmani*, *L*. *obscura* and *L*. *decolor*, respectively, were designed to perform single and multiplex TaqMan qPCR (Table 2). The probes were labeled using different reporter dyes: 6-FAM, HEX, 6-ROXN, Cy5 (Integrated DNA Technologies, Inc., Coralville, IA) and Quasar705 (Biosearch Technologies, Inc., Novato, CA) (Table 3).

**Table 2:** Details of primers.

Target *Liposcelis* species	Primer Name	Primer Sequence (5’-3’)	Length (bp)	GC%	*Tm	**ΔG	^^^any	^^^^3’	Intended use
*Liposcelis brunnea*	BruCo5F	GGGGTTTTAGGGTTTGTGGTA	21	47.2	60.0	0.8	2	2	Endpoint/qPCR multiplex
	BruCo5R	AATGTCGCCAACCATCTAAAG	21	42.9	59.1	0	4	2	Endpoint/qPCR multiplex
	BruCo6F	TTTATGCGATAGGAGCGATTG	21	42.9	60.2	0.5	4	2	Single PCR
	BruCo6R	CATCCAACCCGACAGTAAACA	21	47.7	60.7	1.0	3	0	Single PCR
*Liposcelis bostrychophila*	BosCo7F	CGATCCCTACCGGAGTTAAAG	21	52.4	60.0	0.7	4	1	Endpoint PCR multiplex
	BosCo7R	TGGGCAACAACATAGTATCTATCG	24	41.7	60.3	0.7	6	4	Endpoint PCR multiplex
	BosCo8F	ATGCTCTCAATCGGAGCTCTAG	22	50.0	60.1	0.7	6	4	qPCR multiplex
	BosCo8R	ACTTTAACTCCGGTAGGGATCG	22	50.0	60.7	0.9	4	2	qPCR multiplex
	BosCo9F	TGGGCTAATCTCTCACATCATCT	23	43.5	60.1	0.9	3	3	Single PCR
*Liposcelis decolor*	DecCo10F	CCGGCTTTTGGTATTATTTCAC	22	40.9	59.8	0.7	4	1	Single PCR
	DecCo10R	ATTATCCCCATTACCCCAAA	20	40.0	58.0	0.9	3	0	Single PCR
	DecCo11F	CGAGCTTATTTTACTTCTGCGACT	24	41.7	60.4	0.9	4	1	Endpoint/qPCR multiplex
	DecCo11R	TGATCCATACAACGTAGCTAGTCA	24	41.7	58.9	1.0	6	2	Endpoint/qPCR multiplex
*Liposcelis obscura*	ObsCo12F	GGACAGGGTGGACGGTTTATC	21	57.1	62.8	1.0	3	1	qPCR multiplex
	ObsCo12R	CTGATTCCTGCTAAATGAAGAGAG	24	41.7	58.8	0.9	3	0	qPCR multiplex
	ObsCo13F	AGCTATTGCTCACGGAGGATA	21	47.6	59.0	0	6	4	Endpoint PCR multiplex
	ObsCo13R	CCAATTGCGGACATAGCATAA	21	42.9	60.8	1.0	6	1	Endpoint PCR multiplex
*Liposcelis pearmani*	PeaCo14F	CTGACTTTTTCCCCCTTCACT	21	47.6	59.6	0.9	3	1	qPCR multiplex
	PeaCo14R	GCCAGGGTGTGAGATTCTAAA	21	47.6	59.2	0.9	5	3	Endpoint/qPCR multiplex
	PeaCo15F	TGGTGTGTGAGCAGGTATGGT	21	52.4	61.5	0.8	2	0	Endpoint PCR multiplex

Sequences of *Liposcelis* species specific primers and their thermodynamic features used for endpoint PCR and real-time TaqMan qPCR.

*The melting temperature of the primer calculated using Primer 3;

**Plot ΔG value in plot calculated by mFOLD;

^ The self-complementarity score of the oligo (tendency of oligo to anneal to itself or form a secondary structure) calculated using Primer 3;

^^3’ self-complementarity of the oligo (tendency to form a primer-dimer with itself) calculated using Primer3.

**Table 3:** Details of TaqMan probes.

Target *Liposcelis* species	Probe	Probe Sequence (5’-3’)	Length (bp)	GC%	*ΔG	^^^any	^^^^3’	Reporter dye	Spectra (nm)		Quencher dye	Channel used in Rotor Gene
									EX	EM		
*Liposcelis decolor*	DecCo11P	TGCTGTTCCTACAGGAATCAAGGTTTT	27	41	1.0	8	0	6-FAM	495	520	BHQ2	Green
*Liposcelis brunnea*	BruCo5P	TGTTTACTGTCGGGTTGGATGTGGA	25	48	0.9	3	0	HEX	535	554	ZEN-IABkFQ	Yellow
*Liposcelis obscura*	ObsCo12P	CCTCAGCTATTGCTCACGGAGGA	23	56	0.9	6	4	6-ROXN	575	602	BHQ2	Orange
*Liposcelis bostrychophila*	BosCo8P	GGGCATGGATGTGGATAGACGAGC	24	58	1.0	4	2	Cy5	647	667	BHQ2	Red
*Liposcelis pearmani*	PeaCo14P	AGGGAGTCGGAACCGGGTGAA	21	62	0.8	5	0	Quasar705	690	705	BHQ3	Crimson

*Liposcelis* species specific probes used for multiplex TaqMan qPCR.

*Plot ΔG value in plot calculated by mFOLD;

^ The self-complementarity score of the oligo (tendency of oligo to anneal to itself or form a secondary structure) calculated using Primer 3;

^^3’ self-complementarity of the oligo (tendency to form a primer-dimer with itself) calculated using Primer3; EX is the excitation spectra and EM is the emission spectra.

### Multiplex endpoint PCR

Single PCR reactions with all species specific primer combinations were performed according to conditions and components described by Arif et al. [30]. Multiplex PCR assays were carried out in 50 μl of reaction mixtures containing 25 μl of Multiplex PCR Master Mix (Qiagen), 0.2 μM of each primer, 5 μl of Q-solution (Qiagen), 1 μl of DNA template from each species and nuclease free water (Ambion, Austin, TX) to make up the volume. PCR amplifications were accomplished in an Eppendorf thermal cycler (Eppendorf, Hauppauge, NY). The cycling parameters were: 15 min at 95°C to activate the HotStar *Taq* DNA Polymerase followed by 35 cycles, denaturation at 95°C for 20 sec, annealing at 60°C for 90 sec, extension 72°C for 60 sec and final extension at 72°C for 4 min. Amplified PCR products (20 μl) were electrophoresed and separated in a 2% agarose gel in 1X TAE buffer.

### Multiplex TaqMan qPCR

Multiplex TaqMan qPCR reactions were carried out in 25 μl reaction mixtures containing 12.5 μl of Rotor-Gene Multiplex PCR Master Mix (Qiagen), 0.5 μM of each primer, 0.2 μM of each probe, 1 μl of each template DNA and nuclease free water to make up the volume. Positive (cloned plasmid DNA: carrying the target gene fragment) and negative (non-template; water) controls were encompassed in each TaqMan qPCR amplification. Each qPCR reaction was completed in three replicates and standard deviation was calculated. Cycling parameters were: 95°C for 5 min to activate the HotStar Taq Polymerase followed by 40 rapid cycles at 95°C for 15 sec, and 60°C for 15 sec. In case of SsoFast Probes Supermix (Bio-Rad, Hercules, CA), reactions were carried out in 20 μl mixture containing 10 μl of SsoFast Probes Supermix, 0.5 μM of each primer, 0.2 μM of each probe, 1 μl of each template DNA and nuclease free water to make up the volume. Cycling parameters for SsoFast Probes Supermix were: 95°C for 2 min initial denaturation followed by 40 rapid cycles at 95°C for 5 sec, and 60°C for 30 sec. The assays were performed in a Rotor-Gene 6000 thermocycler and data analysis was achieved using the Rotor-Gene 6000 series software 1.7 (Built 87) (Corbett Research, Sydney, Australia) with auto and manual cycle threshold (Ct) of 0.2. Auto gain optimization was performed before first acquisition and dynamic tube-based normalization was used.

### Multi target artificial positive control (APC)

**Fig 1:**
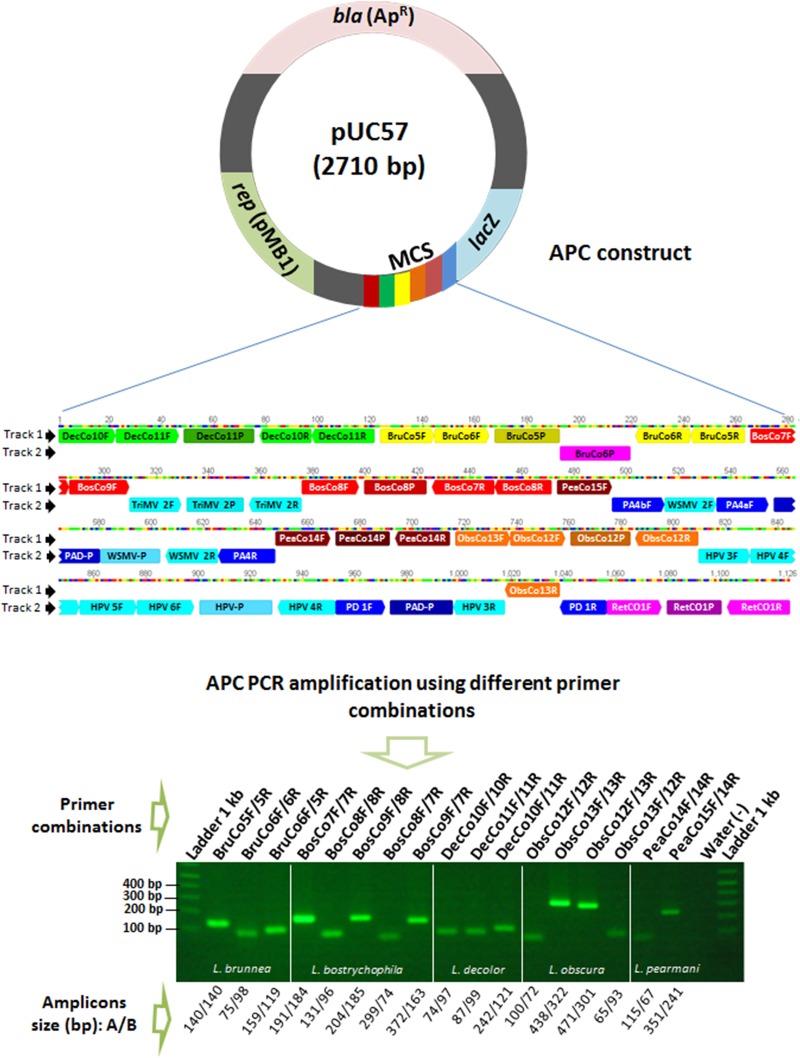
The multi-target artificial positive control (APC). Top, APC made by a custom synthesized DNA insert containing tandems of forward, reverse and probe complement priming sequences ligated into the multiple cloning site (MCS) in the vector pUC57. Each amplified PCR product using APC has a unique identifiable sequence. Bottom, the product sizes of the targets differ from the product size and sequences amplified using genomic DNA. A/B, where A is amplicon size generated using genomic DNA of target species and B is amplicon size generated using APC (shown in picture). Different colors at track 1 are the indications of reporter dyes wavelengths (excitation and emission spectra) detected by different channels of real-time qPCR for particular species of *Liposcelis* as shown in Table 3. Primers and probes sequences indicated by track 1 were used in this study. Primers and probes sequences indicated by track 2 are from fungi, viruses and insect and were not used in this study. Lists of primer and probe sequences are given in Tables 2 and 3, respectively.

A multi-target APC, generated synthetically (GenScript USA Inc, Piscataway, NJ) was 1126 bp long and composed of tandems of both sense and anti-sense primers and probes including the target sequences of the five *Liposcelis* species (accession number GenBank:KC555272) in addition to primer and probe sequences of several other phytopathogens (Fig 1). The array of synthetic oligonucleotides was inserted in the multiple cloning site of cloning vector pUC57 (NCBI accession number GenBank:Y14837). PCR generated products obtained with primer sets BruCo6F/BruCo5R (159 bp), BosCo9F/BosCo7R (372 bp), DecCo10F/DecCo11R (242 bp), ObsCo12F/ObsCo13R (471 bp) and PeaCo15F/PeaCo14R (351 bp) were circularized within pCR 2.1-TOPO vector and cloned using TOP10F´ One Shot Chemically Competent cells (TOPO-TA Cloning kit; Invitrogen). The sequences obtained from these cloned DNAs were analyzed *in silico* for specificity and accuracy.

### Sensitivity assays

The sensitivity and efficiency of each primer set used in endpoint and multiplex TaqMan qPCR were assessed after 10-fold serial dilutions of the developed APC carrying all the target sequences of each primer and probe of the five *Liposcelis* species. Dilutions of purified APC DNA ranged from 1 ng to 1 fg per reaction. Both endpoint and multiplex TaqMan qPCR assays were also tested to amplify the DNA extracted from an individual insect.

## Results

### Primer and probe specificity

**Fig 2:**
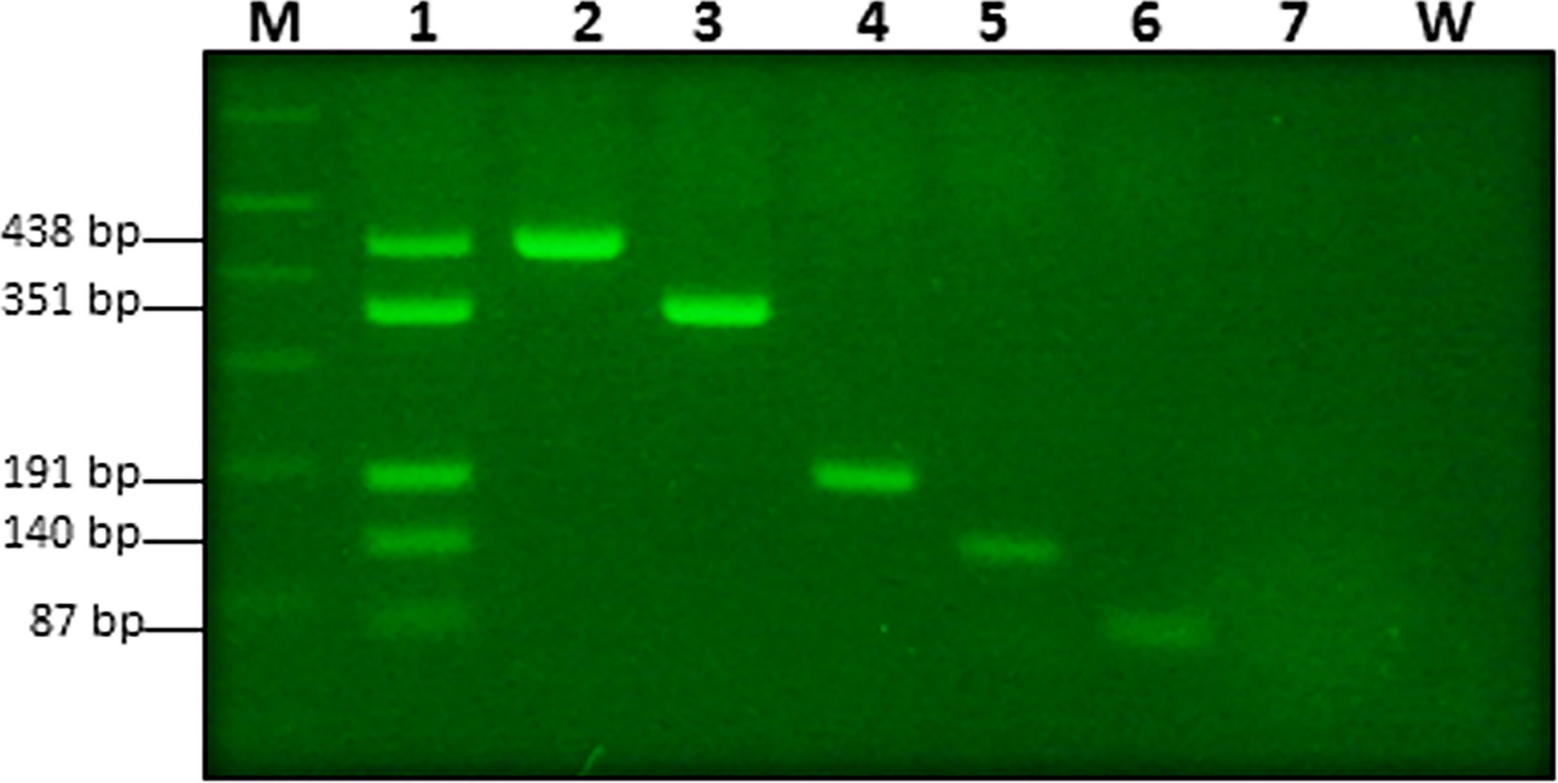
Multiplex endpoint PCR. Assay was performed with insect genomic DNA and primer sets ObsCo13F/13R (438 bp), PeaCo15F/14R (351 bp), BosCO7F/7R (191 bp), BruCo5F/5R (140 bp), and DecCo11F/11R (87 bp) for *L*. *obscura*, *L*. *pearmani*, *L*. *bostrychophila*, *L*. *brunnea* and *L*. *decolor*, respectively. Lane 1: PCR reaction with all five species genomic DNA. Lanes 2–6: single species genomic DNA *viz*. *L*. *obscura*, *L*. *pearmani*, *L*. *bostrychophila*, *L*. *brunnea* and *L*. *decolor*, respectively. Lanes M and W are 1 kb ladder and non-template control (water), respectively.

The use of insect CO1 universal primer combinations allowed the amplification of unknown regions of the CO1 gene of *L*. *decolor*, *L*. *bostrychophila*, *L*. *pearmani* and *L*. *obscura*. Specific primers (Table 2) and probes (Table 3) were then designed from the amplified and sequenced CO1 gene regions. These forward and reverse primers were tested in combination against targeted *Liposcelis* species. The primers were selected based on the product size of each amplified combination (Table 2). For example, primer set PeaCo14F/PeaCo14R, specific for *L*. *pearmani*, generated an amplicon of 115 bp, which was ideal for single or multiplex TaqMan qPCR, but this set was not suitable for endpoint multiplex PCR when product size was evaluated among other four amplicons. When PeaCo14F was replaced with PeaCo15F, the amplicon size increased to 351 bp, a size appropriate for multiplex endpoint PCR (Figs 1 and 2).

The specificity of the twenty primer (Table 2) was tested *in silico* and *in vitro*. The alignment of primer sequences against the GenBank database using BLASTn showed that none of the primers matched with 100% query coverage and 100% identity, because they are newly contributed CO1 gene sequences. However, the primers designed specifically for *L*. *brunnea* matched (100%) with accession GenBank:GU569291, as expected. The *in vitro* specificity assays were performed (using PCR) with each primer set against a panel of near-neighbors including *L*. *inquilinus*, *L*. *patruelis*, *L*. *reticulatus*, *L*. *brunnea*, *L*. *bostrychophila*, *L*. *decolor*, *L*. *rufa*, *L*. *entomophila*, *L*. *paeta*, *L*. *fusciceps*, *L*. *obscura*, *L*. *pearmani*, and *L*. *corrodens* (results not shown). No cross reactivity was observed with non-target species in the exclusivity panel. All primer sets amplified only the corresponding *Liposcelis* species and generated the expected PCR product size with genomic DNA and APC (Fig 1). All primer sets also yielded appropriate negative results when tested for cross reactivity against DNA of the insect *Homalodisca vitripennis* (Germar) (an out-group) and of cracked wheat grain (a host).

### Multiplex endpoint PCR

**Fig 3:**
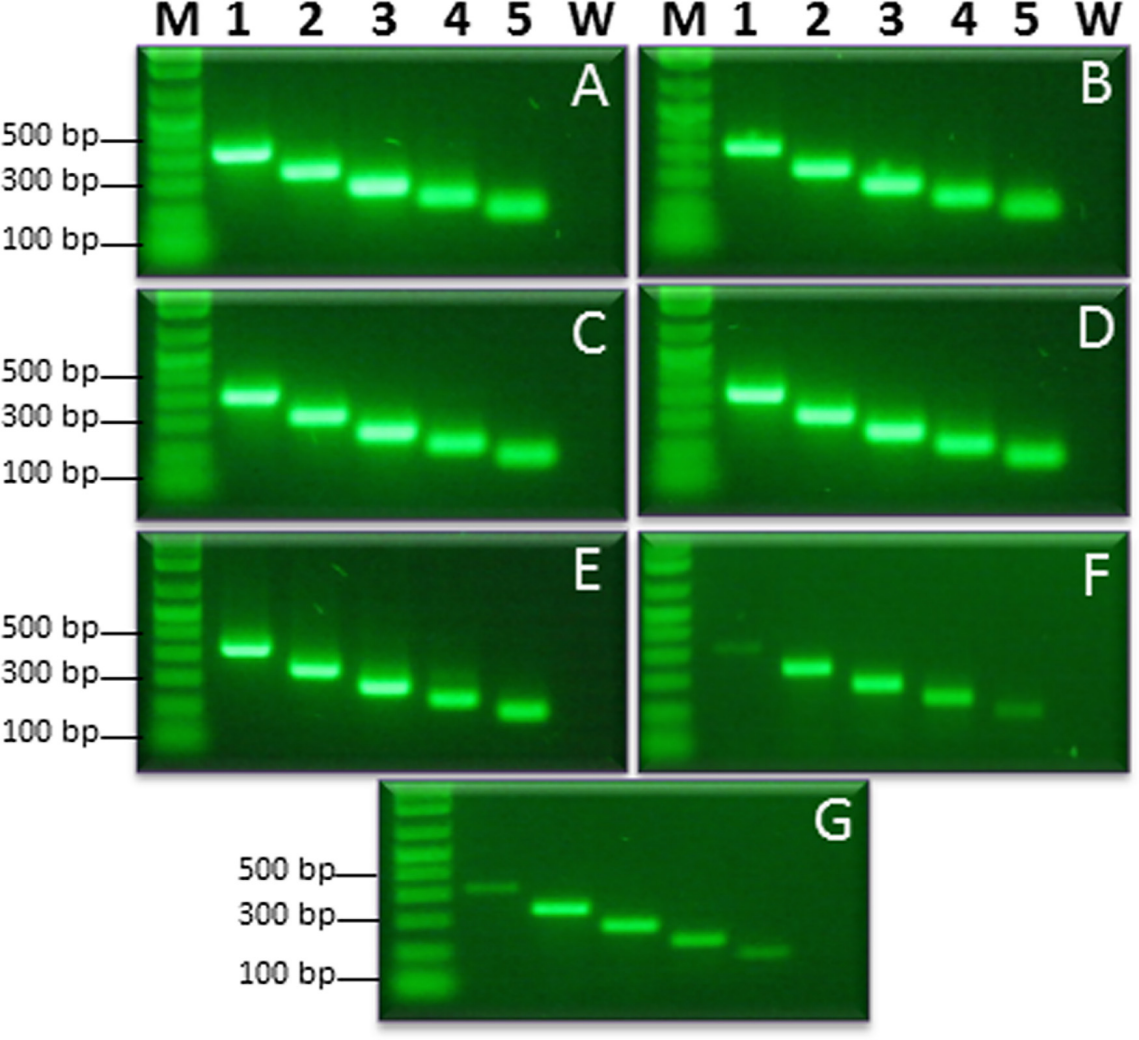
Endpoint PCR sensitivity assays with individual primer set. Endpoint PCR performed with individual primer set using 10-fold serially diluted multi target artificial positive control (APC) starting at (A) 1 ng, (B) 100 pg, (C) 10 pg, (D) 1000 fg, (E) 100 fg, (F) 10 fg, and (G) 1 fg. Lane 1 to 5 are primer sets, ObsCo13F/13R (322 bp), PeaCo15F/14R (241 bp), BosCO7F/7R (184 bp), BruCo5F/5R (140 bp), and DecCo11F/11R (99 bp), respectively. Lanes M is a 1 kb ladder and W is a non-template control (water).

Multiplex endpoint PCRs for detection of *L*. *obscura*, *L*. *pearmani*, *L*. *bostrychophila*, *L*. *brunnea* and *L*. *decolor* were performed using primer sets ObsCo13F/13R, PeaCo15F/14R, BosCO7F/7R, BruCo5F/5R, and DecCo11F/11R, which amplified products of 438-, 351-, 191-, 140-, and 87-bp, respectively (Fig 2 and S2 Fig). Each primer set specifically amplified the corresponding target *Liposcelis* species. The sensitivity of each primer set was checked with serially diluted APCs. Each primer set detected as little as 1 fg of target APC (Fig 3). The developed multiplex endpoint PCR gave positive results when tested against crude genomic DNA extracted from an individual insect (S2 Fig). No cross reactivity was observed against the non-targeted and/or closely related species of genus *Liposcelis*.

### Multiplex TaqMan qPCR

**Fig 4:**
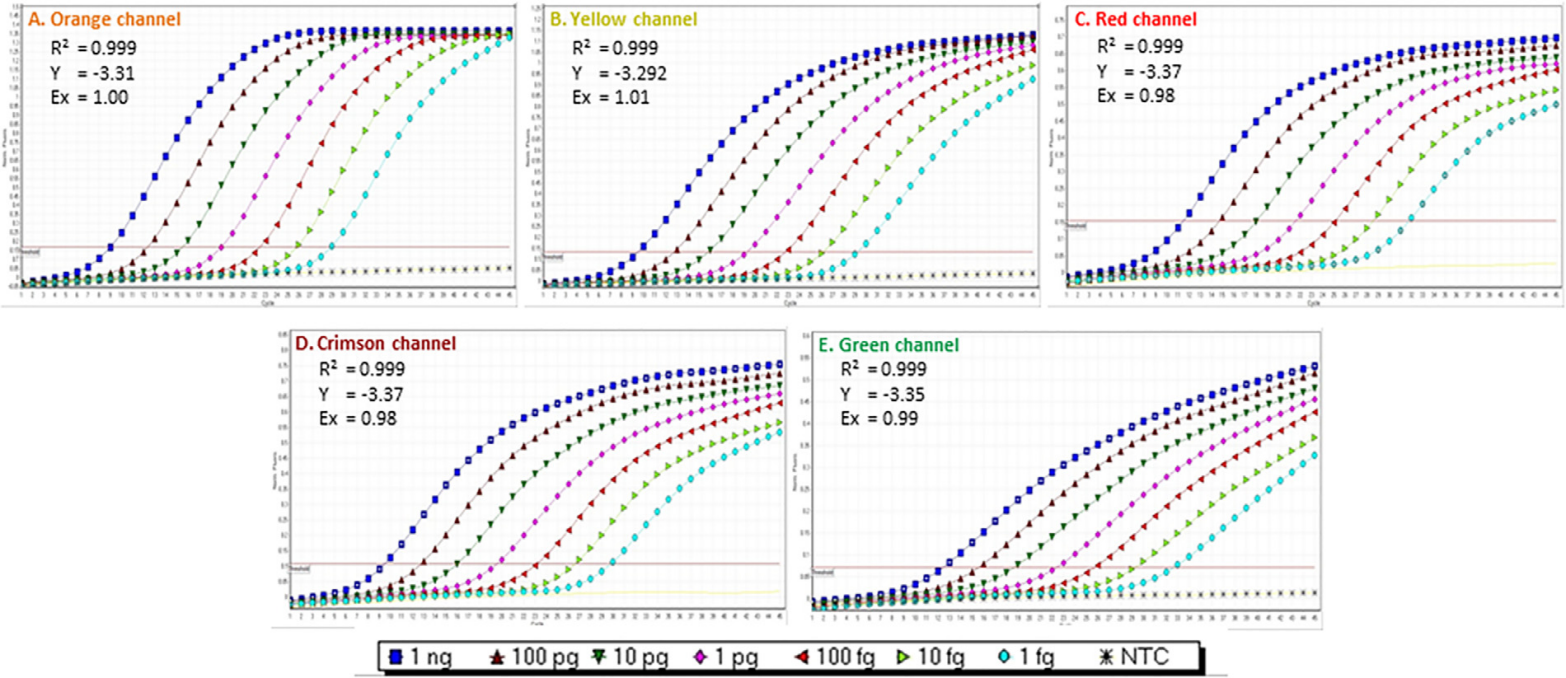
Multiplex TaqMan qPCR sensitivity assay. Assay was performed with 10-fold serially diluted multi target artificial positive control (APC) from 1 ng to 1 fg using primer and probe sets (A) ObsCo12F/12R/12P (72 bp), (B) BruCo5F/5R/5P (140 bp), (C) BosCo8F/8R/8P (96 bp), (D) PeaCo14F/14R/14P (67 bp), and (E) DecCo11F/11R/11P (99 bp). Different channels *viz*. orange, yellow, red, crimson and green corresponding to the different reporter dye (excitation/emission spectra in nm) *viz*. 6-ROXN (575/602), HEX (535/554), Cy5 (647/667) and Quasar705 (690/705) and 6-FAM (495/520), respectively.

**Fig 5:**
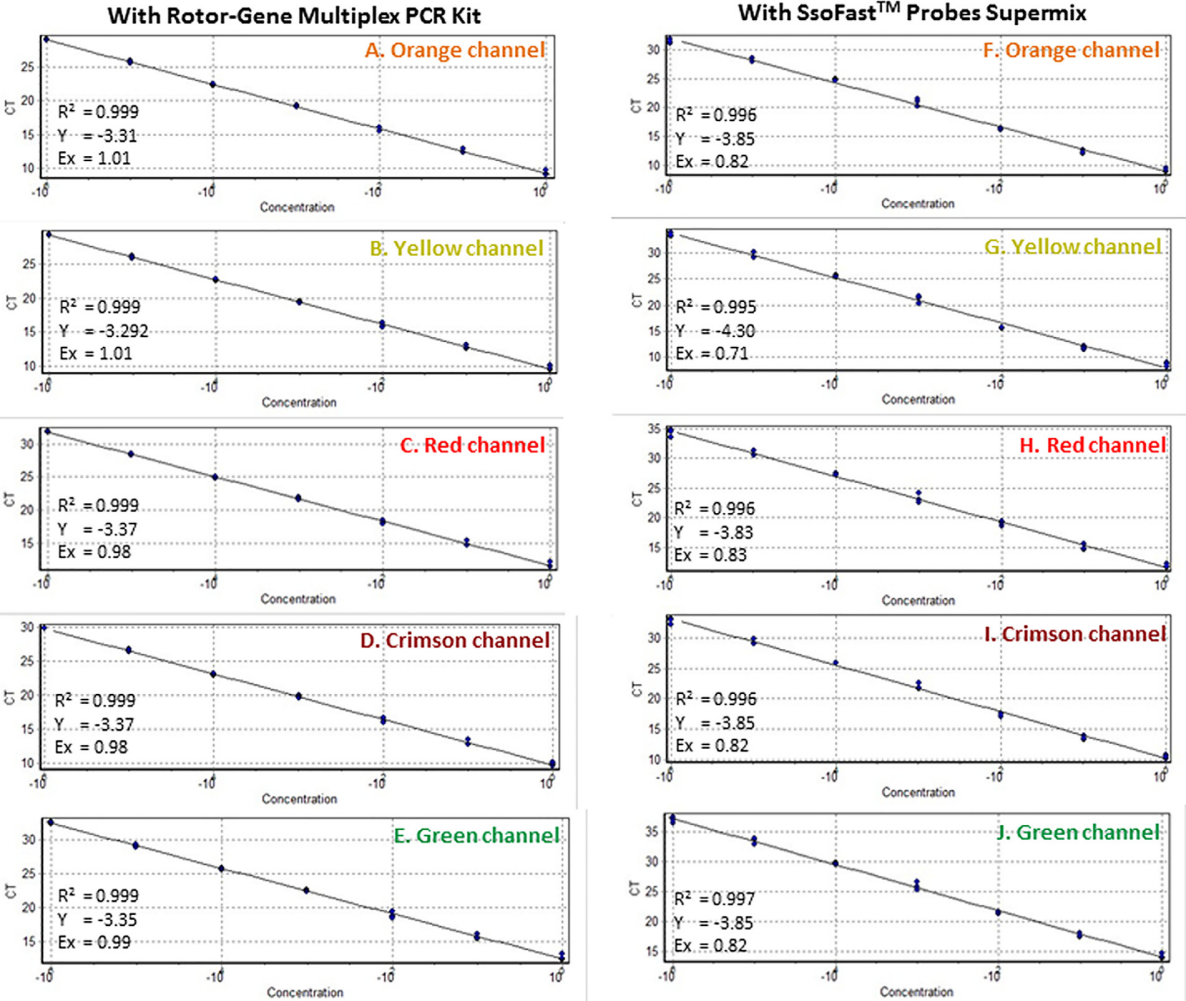
Multiplex TaqMan qPCR standard graphs. Standard graphs were generated using 10-fold serially diluted multi target artificial positive control (APC) from 1 ng to 1 fg using the primer and probe sets BruCo5F/5R/5P (140 bp), BosCo8F/8R/8P (96 bp), DecCo11F/11R/11P (99 bp), ObsCo12F/12R/12P (72 bp) and PeaCo14F/14R/14R (67 bp). Different channels *viz*. orange, yellow, red, crimson and green corresponding to different reporter dye (excitation/emission spectra in nm) *viz*. 6-ROXN (575/602), HEX (535/554), Cy5 (647/667) and Quasar705 (690/705) and 6-FAM (495/520).

The product sizes resulting from the use of primer sets in multiplex TaqMan qPCR ranged from 87 bp to 140 bp (Fig 1). The excitation/emission spectra (nm) of each probe labeled with 6-FAM (495/520 nm), HEX (535/554 nm), 6-ROXN (575/602 nm), Cy5 (647/667 nm) and Quasar705 (690/705 nm) was different from those of the others, avoiding the overlapping of spectra that could be detected by a corresponding channel leading to false positive results. All channels, whether green, yellow, orange, red or crimson, specifically detected the fluorescence that resulted from its corresponding reporter dye (Table 3). The efficiency of multiplex TaqMan qPCR was evaluated by performing a sensitivity assay with 10-fold serially diluted APC carrying the target sequences of all primer and probe sets (Tables 2 and 3, Figs 4 and 5). The primer and probe sets ObsCo12F/12R/12P; BruCo5F/5R/5P; BosCo8F/8R/8P; PeaCo14F/14R/14P; and DecCo11F/11R/11P, detected down to 1 fg of APC (Table 4, Fig 4) with Ct values of 29.09, 29.36, 31.83, 29.87 and 32.62, respectively. The low Ct value and ideal linear correlation (R^2^; 0.999), slope (Y; -3.37 to -3.29) and reaction efficiency (Ex; 0.98–1.01) using the Rotor-Gene Multiplex qPCR kit showed high efficiency and accuracy for each primer and probe set in multiplex TaqMan qPCR (Table 4). A low standard deviation among replicates of each dilution within each primer and probe set (Table 4) also indicates the assays are highly accurate. When sensitivity was assessed using SsoFast Probe Mix (a master mix recommended for single TaqMan qPCR) as little as 1 fg of plasmid DNA (Table 4) was detected, indicating good compatibility and thermodynamics. However, reaction efficiency was not as good as using the Rotor-Gene Multiplex qPCR kit (Table 4, Fig 5).

**Table 4:** Comparative multiplex TaqMan qPCR sensitivity assays value.

	Template conc. per reaction	Ct(SD) value of corresponding channel using Rotor-Gene Multiplex qPCR kit					Ct(SD) value of corresponding channel using SsoFast Probe Supermix				
		Orange	Yellow	Red	Crimson	Green	Orange	Yellow	Red	Crimson	Green
**R^2^**		0.999	0.999	0.999	0.999	0.999	0.996	0.995	0.996	0.996	0.997
**Y**		-3.31	-3.29	-3.37	-3.37	-3.35	-3.85	-4.30	-3.83	-3.85	-3.85
**Ex**		1.01	1.01	0.98	0.98	0.99	0.82	0.71	0.83	0.82	0.82
*Ct(SD) values	1 ng	9.32(0.36)	9.68(0.36)	11.71(0.37)	9.76(0.32)	12.66(0.43)	9.13(0.36)	8.56(0.43)	11.93(0.29)	10.41(0.31)	14.20(0.41)
	100 pg	12.52(0.29)	12.84(0.28)	14.92(0.39)	12.97(0.35)	15.73(0.37)	12.11(0.11)	11.63(0.18)	14.78(0.17)	13.33(0.15)	17.46(0.11)
	10 pg	15.70(0.30)	16.00(0.34)	18.19(0.31)	16.21(0.35)	18.91(0.50)	16.28(0.16)	15.70(0.11)	19.28(0.19)	17.55(0.03)	21.58(0.11)
	1 pg	19.17(0.13)	19.39(0.12)	21.74(0.17)	19.75(0.19)	22.55(0.13)	20.89(0.56)	21.12(0.71)	23.30(0.76)	21.97(0.53)	25.86(0.60)
	100 fg	22.42(0.12)	22.65(0.13)	24.98(0.17)	23.07(0.14)	25.73(0.16)	24.74(0.11)	25.59(0.17)	27.26(0.15)	25.90(0.07)	29.68(0.15)
	10 fg	25.84(0.21)	26.09(0.17)	28.48(0.17)	26.59(0.20)	29.23(0.20)	28.34(0.40)	29.80(0.59)	31.03(0.48)	29.57(0.50)	33.47(0.50)
	1 fg	29.09(0.10)	29.36(0.08)	31.83(0.08)	29.87(0.02)	32.62(0.11)	31.48(0.38)	33.43(0.36)	34.25(0.69)	32.78(0.47)	36.85(0.51)
	NTC	-	-	-	-	-	-	-	-	-	-

Average Ct values of sensitivity assays using 10-fold serial dilutions of a multi-target artificial positive control (APC) from 1 ng to 1 fg with primer and probe sets BruCo5F/5R/5P, BosCo8F/8R/8P, DecCo11F/11R/11P, ObsCo12F/12R/12P and PeaCo14F/14R/14P.

R^2^, linear correlation; Y, slope; Ex, reaction efficiency; SD, standard deviation; the Ct (threshold cycle) values are average of three replicates.

A small difference in Ct values was observed when multiplex TaqMan qPCR reactions were performed in separate tubes using genomic DNA extracted from either a single *Liposcelis* species (DNA from individual species was added in a multiplex qPCR) or a mixture of all the five-species of *Liposcelis* (DNA from all species was added in a single multiplex qPCR). The Ct values (individual species/ targeted species all together) in multiplex TaqMan qPCR corresponding to different channels were orange (20.06/23.37), yellow (21.64/21.85), red (20.76/20.67), crimson (27.09/31.53), and green (23.64/23.37). The reaction efficiency (0.95–1.0), linear correlation (0.999), and slope (-3.44 to -3.32) of all other channels (Table 5) were within an optimal range. A repeat experiment using new extracted genomic DNA of all targeted insects produced the same results (Table 5). DNA isolated from an individual insect was detected using this multiplex TaqMan qPCR.

**Table 5:** Multiplex TaqMan qPCR assays with *Liposcelis* genomic DNA.

	Template conc. per reaction	Ct(SD) value of corresponding channel with genomic DNA					Ct(SD) value of corresponding channel with genomic DNA (performed after 8 months)				
		Orange	Yellow	Red	Crimson	Green	Orange	Yellow	Red	Crimson	Green
**R^2^**		0.999	0.999	0.999	0.999	0.999	0.999	0.999	0.999	0.999	0.999
**Y**		-3.319	-3.364	-3.384	-3.444	-3.433	-3.370	-3.178	-3.368	-3.266	-3.388
**Ex**		1.00	0.98	0.97	0.95	0.96	0.98	1.06	0.98	1.02	0.97
**Ct(SD) values**	1 ng	9.01(0.05)	10.55(0.06)	11.12(0.09)	9.52(0.05)	13.61(0.03)	9.38(0.02)	9.56(0.27)*	11.39(0.15)	10.33(0.17)	12.68(0.20)
	100 pg	12.29(0.05)	14.03(0.06)	14.56(0.04)	13.08(0.02)	17.02(0.11)	12.75(0.04)	11.66(0.02)	14.72(0.11)	13.67(0.04)	16.07(0.06)
	10 pg	15.64(0.04)	17.28(0.06)	17.86(0.06)	16.41(0.08)	20.48(0.07)	16.12(0.03)	14.84(0.02)	18.13(0.08)	16.86(0.05)	19.45(0.09)
	With single species	20.06(0.08)	21.64(0.13)	20.76(0.11)	27.09(0.17)	23.64(0.41)	24.29(0.25)	18.54(0.19)	19.68(0.02)	25.67(0.13)	22.65(0.21)
	With all five species	23.37(0.32)	21.85(0.15)	20.67(0.15)	31.53(1.23)	23.37(0.33)	22.27(0.05)	17.57(0.02)	19.19(0.01)	28.34(0.87)	22.30(0.12)
	NTC	-	-	-	-	-	-	-	-	-	-

Average Ct values using genomic DNA of *Liposcelis brunnea*, *L*. *bostrychophila*, *L*. *decolor*, *L*. *obscura* and *L*. *pearmani* with primer and probe sets BruCo5F/5R/5P, BosCo8F/8R/8P, DecCo11F/11R/11P, ObsCo12F/12R/12P and PeaCo14F/14R/14P, respectively.

*Due to error in fluorescence, this value has not been included in analysis; R^2^, linear correlation; Y, slope; Ex, reaction efficiency; SD, standard deviation; the Ct values are average of three replicates; Orange, yellow, red, crimson and green color corresponding to *L*. *obscura*, *L*. *brunnea*, *L*. *bostrychophila*, *L*. *pearmani* and *L*. *decolor*, respectively.

### Multi-target artificial positive control

A unique multi-target APC was designed, with all the target primer and probe sequences, to amplify a product for each primer set that would have a size compatible for different PCR techniques (Fig 1). The size difference in the amplified products allows visualization of products of the APC that can be used distinctly for endpoint and real-time qPCR. The probe sequences were adjusted to be between the forward and reverse primers for qPCR (Fig 1). The developed APC also carries sense and anti-sense primer sequences of other pathogens including the viruses High plains virus, Wheat streak mosaic virus and Triticum mosaic virus, and the fungi *Pythium aphanidermatum* and *Pythium deliense*. To check the reproducibility of assays using the APC, PCR amplifications using all 17 *Liposcelis* primer combinations were repeated three times. All results obtained were accurate and reproducible (Fig 1). The APC was used in endpoint PCR and to generate standard graphs for multiplex qPCR (Figs 3–5). The multiplex TaqMan qPCR showed 0.999 linear correlations, -3.37 to -3.292 slopes and 0.98 to 1.01 reaction efficiencies.

## Discussion

We have developed and validated two methods for the simultaneous detection and discrimination of *L*. *brunnea*, *L*. *decolor*, *L*. *bostrychophila*, *L*. *pearmani* and *L*. *obscura* using multiplex versions of both endpoint and real-time TaqMan qPCR. The developed primer sets can also be used for species-specific single endpoint or real-time TaqMan qPCR. Furthermore, we have designed and developed a single, unique multi-target and clonable APC that mimics these five species of genus *Liposcelis*.

In pest management, biosecurity, quarantine and routine diagnostics, accuracy, reliability and sensitive discriminatory capabilities are important [35, 47]. New segments of the *Liposcelis* CO1 region were amplified using endpoint PCR from *L*. *decolor*, *L*. *bostrychophila*, *L*. *pearmani* and *L*. *obscura* using a combination of previously reported primers. The generated sequences were used to design species specific primers and probes from signature diagnostic targets. A conserved region of the COI gene which is reliable for identification purposes, was previously used for the detection of *L*. *entomophila*, *L*. *corrodens* and *L*. *reticulatus* [30, 32, 48–50] and led us to use a primer combination approach based on previously reported universal primers for insects (S1 Fig), which facilitated the amplification of the unknown CO1 gene regions of *L*. *decolor*, *L*. *bostrychophila*, *L*. *pearman* and *L*. *obscura*. This approach would be suitable for the amplification of CO1 or unknown gene regions of other species. Subsequently, specific primers and probes for multiplex endpoint and real-time TaqMan qPCR were designed for detection and discrimination of the five *Liposcelis* species. All primers and probes were designed to have minimal secondary structure, delta G values equal or close to zero for increased sensitivity and compatibility, and reduced thermodynamic interference during PCR [45]. A second approach using target specific primers was used to determine the best reverse and forward primer sets with maximum PCR yield or fluorescence, a capability of amplifying PCR products with different sizes, and good compatibility during multiplex endpoint PCR and real-time TaqMan qPCR for better discrimination of the five *Liposcelis* targets (Table 2 and Fig 1). Previously, Arif et al. [36] used the primer combination approach to develop a primer and probe set having broad range detection capabilities for High plains virus variants.

All the primer sets that were developed are highly specific for their corresponding targets and no cross amplification was detected. All primer sets showed high specificity in multiplex endpoint PCR (Fig 2). Each individual primer set used in multiplex endpoint PCR detected as little as 1 fg of plasmid DNA (APC) carrying the multi-targets for all the primer sets (Fig 3). Saccaggi et al. [51] developed multiplex endpoint PCR for mealybug species (Hemiptera: Pseudococcidae) *Planococcus ficus*, *Planococcus citri* and *Pseudococcus longispinus* and reported high accuracy.

The described qPCR assays can be performed simultaneously (multiplex) and individually using a Rotor-Gene 6000 thermocycler. Different florescent reporter dyes (6-FAM, HEX, 6-ROXN, Cy5 and Quasar705) were selected based on their wavelength to avoid overlapping of wavelengths, which could lead to false positives. The multiplex TaqMan qPCR detected down to 1 fg of APC using Sso Fast Probes Master Mix, which is intended for single qPCR reactions. This result confirmed the high accuracy and compatibility that exists among the primers and probes when performing in multiplex qPCR. The developed assays are capable of detecting and discriminating each target species from the other species of genus *Liposcelis* that were used. This is the first published study on detection and discrimination of stored-grain pest species of any kind using multiplex qPCR methods.

The four main pest species of psocids worldwide are *L*. *bostrychophila*, *L*. *decolor*, *L*. *entomophila*, and *L*. *paeta*. The method we have developed includes only two of these species, namely, *L*. *bostrychophila* and *L*. *decolor*. Given the method we have developed, similar research can be conducted to enable simultaneous identification of the aforementioned four main pest psocid species. Moreover, the APC can be modified to insert the new complement sequences of primers and/or probes designed for specific identification of *L*. *entomophila* and *L*. *paeta*.

The positive controls were essentially developed for assessment of PCR reliability. These controls are challenging to obtain for rare insects or microbes that are exotic and/or emerging pests that pose a potential biosafety risk as regulated insects or infectious pathogens. A functional, clonable multi-target, synthetic, and artificial positive control, custom made of synthetic DNA inserts containing tandems of forward and reverse complement priming sequences, was designed *de novo* and inserted and circularized into a plasmid vector. The product size of amplicons generated from APC with each primer set can vary from amplicons generated using genomic DNA. However, the amplicons size for APCs can be determined at the time of the APC design (Fig 1) which can facilitate discrimination between amplicons generated using genomic DNA and APC, if cross contamination occurred. Moreover, each PCR product generated using APC has a unique identifiable sequence. For example, primer set PeaCO14F and PeaCo14R generate a 115 bp amplicon with genomic DNA of *L*. *pearmani* and 67 bp using APC (Fig 1). The suitability of customized synthetic DNA was demonstrated *in silico* by analyzing the thermodynamics of the selected primers and *in vitro* by PCR [37].

This approach can be used for incorporating hundreds of primer and probe sequences in a clone to use as positive control for a large number of insects, pathogens and other target of interest. The use of this APC will speed-up PCR processing, and will increase accuracy and reliability, minimize costs and will remove the biosafety risks associated with *in vivo* positive controls. This kind of positive control can also be shipped or exchanged among different national and regional diagnostic laboratories, and can be used as a reference for multiple PCR assays where insect and/or pathogen are regulated due to geographical distribution.

## Supporting Information

S1 Fig Location of universal primers within CO1 gene region. Location of different reported universal CO1 primers within the CO1 gene region used to amplify the CO1 region of five species of genus *Liposcelis*.
(TIF)

S2 Fig Endpoint multiplex PCR with individual insect crude DNA. Endpoint multiplex PCR performed with individual insect crude DNA and primer sets ObsCo13F/13R (438 bp), PeaCo15F/14R (351 bp), BosCO7F/7R (191 bp), BruCo5F/5R (140 bp), and DecCo11F/11R (87 bp) for *L*. *obscura*, *L*. *pearmani*, *L*. *bostrychophila*, *L*. *brunnea* and *L*. *decolor*, respectively. Lane 1: PCR reaction with all five species genomic DNA. Lane 2–6: Single species genomic DNA *viz*. *L*. *obscura*, *L*. *pearmani*, *L*. *bostrychophila*, *L*. *brunnea* and *L*. *decolor*, respectively. Lane M and W are 1 kb ladder and non-template control (water), respectively.
(TIF)
