# Supplementary figures and images for: Array of Synthetic Oligonucleotides to Generate Unique Multi-Target Artificial Positive Controls and Molecular Probe-Based Discrimination of Liposcelis Species
Source: PLoS One. 2015 Jun 18;10(6):e0129810. doi: 10.1371/journal.pone.0129810 (PMC4472718; doi:10.1371/journal.pone.0129810)

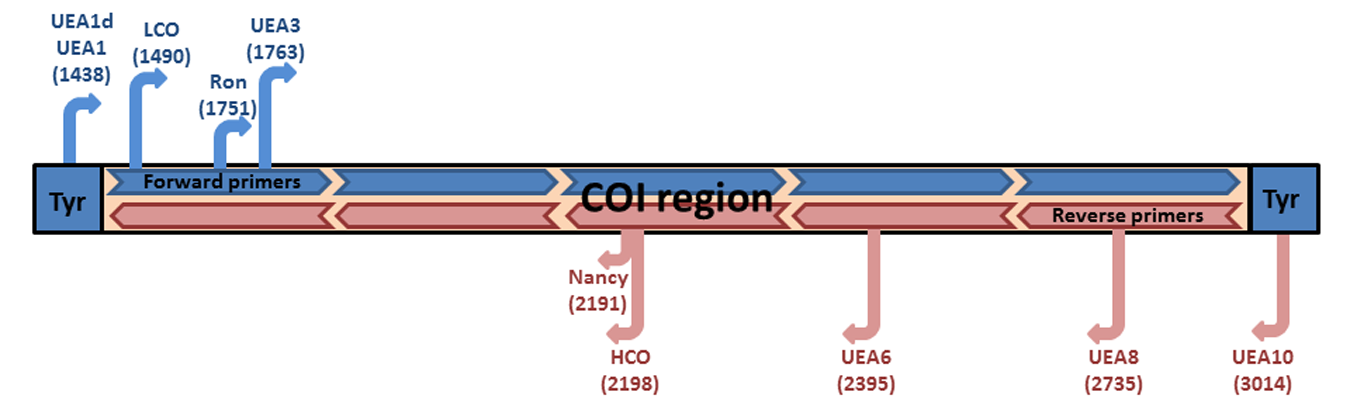

Supplement: S1 Fig — Location of different reported universal CO1 primers within the CO1 gene region used to amplify the CO1 region of five species of genus Liposcelis. (TIF) [file pone.0129810.s001.tif]

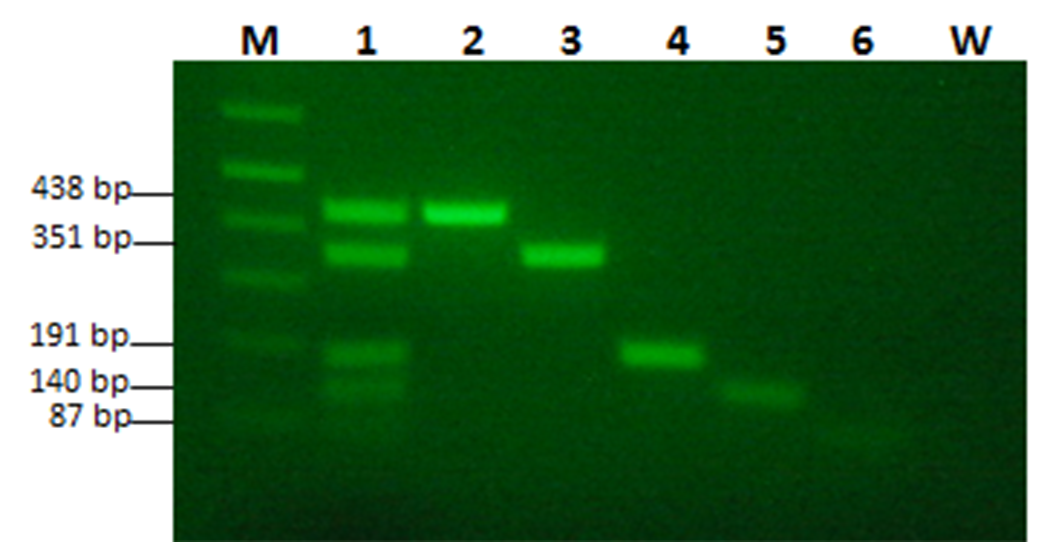

Supplement: S2 Fig — Endpoint multiplex PCR performed with individual insect crude DNA and primer sets ObsCo13F/13R (438 bp), PeaCo15F/14R (351 bp), BosCO7F/7R (191 bp), BruCo5F/5R (140 bp), and DecCo11F/11R (87 bp) for L. obscura, L. pearmani, L. bostrychophila, L. brunnea and L. decolor, respectively. Lane 1: PCR reaction with all five species genomic DNA. Lane 2–6: Single species genomic DNA viz. L. obscura, L. pearmani, L. bostrychophila, L. brunnea and L. decolor, respectively. Lane M and W are 1 kb ladder and non-template control (water), respectively. (TIF) [file pone.0129810.s002.tif]
